# Single-Cell Sequencing Reveals PD-L1-Mediated Immune Escape Signaling in Lung Adenocarcinoma

**DOI:** 10.7150/jca.103656

**Published:** 2025-01-27

**Authors:** Anbing Zhang, Jianping Liang, Xiaoli Lao, Xiuqiong Xia, Siqi Li, Shengming Liu

**Affiliations:** 1 Department of Pulmonary and Critical Care Medicine, The First Affiliated Hospital of Jinan University, Guangzhou 510630, China.; 2 Department of Pulmonary and Critical Care Medicine, Zhongshan People's Hospital, Zhongshan 528403, China.; 3 Graduate School, Guangdong Medical University, Zhanjiang 524023, China.

**Keywords:** Lung adenocarcinoma, Immune checkpoint, PD-L1, Single-cell sequencing, Molecular marker

## Abstract

**Background:** Lung cancer has the highest mortality rate among all cancers, for which immunotherapy can frequently lead to drug resistance. To understand the molecular mechanisms behind immune escape in patients with lung cancer and develop predictive and therapeutic targets, we carried out analytical experiments using single-cell sequencing.

**Methods:** We collected eight tumor tissue samples from eight patients with lung adenocarcinoma and categorized them based on the positive reactions for programmed cell death ligand 1 (PD-L1) expression levels. Single-cell sequencing analysis was employed to create a comprehensive cellular landscape. Uniform Manifold Approximation and Projection was used to show the proportion of immune and endothelial cells, along with a map depicting the distribution of different cell types. Cells were subdivided according to molecular markers; the subpopulations were grouped based on PD-L1 levels and tumor marker-positive reactions. The correlation between the occurrence of the PD-L1 reaction and the response time of immune cells was explored; differential gene expression between the groups was elucidated. Finally, quantitative polymerase chain reaction (qPCR) was used to examine the relationship between key differentially expressed genes and PD-L1 immune escape checkpoint response.

**Results:** A total of 58,810 single cells were analyzed, identifying seven distinct cell types. In the PD-L1-positive sample group, B cells, astrocytes, endothelial cells, outer skin cells, and tissue stem cells were present in higher proportions, whereas T and dendritic cells were the main cells in the PD-L1-negative sample group. According to the molecular markers, the seven cell types were divided into 17 cell clusters, with one cluster classified as tumor cells, showing PD-L1 positivity. Eleven molecular markers with different expression levels were simultaneously screened (NAPSA, MUC1, WFDC2, MYO6, LYZ, IGHG4, IGLL5, IGHM, IGKC, AQP3, and IGFBP7), and their association with the PD-L1/PD-1 immune escape axis response was confirmed by qPCR.

**Conclusion:** Our study suggests that PD-L1-mediated immune escape may occur at a later stage of tumor progression, involving both PD-L1-positive and negative immune cells. Additionally, we identified 11 differentially expressed genes that could provide insights into the potential mechanisms of immune escape in patients with lung cancer. These findings offer promising molecular targets for the detection and treatment of immune escape in clinical settings.

## Introduction

Lung cancer is the most common malignant tumor of the lungs and bronchi. According to statistics from the American Cancer Society (2020-2021), there were an estimated 2.2 million new cases of lung cancer and 1.8 million deaths worldwide 1,2. In the United States alone, it was estimated that 235,760 new cases of lung cancer would be diagnosed in 2020, accounting for 12.4% of all malignant tumors. The number of deaths was expected to reach 131,800, representing 21.6% of all cancer-related deaths and making lung cancer the leading cause of cancer mortality in the U.S. 3. Approximately 84% of lung cancer cases are non-small cell lung cancer, with lung adenocarcinoma (LUAD) being the most common histological subtype, accounting for 50% of lung cancer cases 4. The prognosis of LUAD is generally poor, with limited median survival. Early-stage LUAD is primarily treated with surgery, while mid-to-late stages are treated with chemotherapy and immunotherapy. Although many patients have benefited from advances in targeted therapy and immunotherapy, certain challenges remain, including differences in individual treatment sensitivity and the development of tumor-acquired drug resistance.

Immunotherapy for LUAD includes immune checkpoint inhibitors (ICIs), tumor vaccines, cytokines, and chimeric antigen receptor T-cell immunotherapy, with ICIs receiving significant research attention in recent years 5-7. ICIs, which disrupt the body's immune system and reactivate its defense role, have been increasingly used for a variety of cancer types over the past decade. Current ICI studies mainly focus on cytotoxic T lymphocyte-associated antigen-4 (CTLA-4), programmed cell death 1 (PD-1), and programmed cell death-ligand 1 (PD-L1). PD-1/PD-L1 specifically regulates T cell activity within tumors, thereby limiting autoimmunity, while CTLA-4 modulates the early activation of both naïve and memory T cells 5.

PD-L1 is widely expressed in various cell types, including tumor cells, monocytes, macrophages, natural killer cells, dendritic cells (DCs), and activated T cells. PD-1 is an immune checkpoint protein expressed in T cells. When PD-1 expressed in activated T cells interacts with PD-L1 expressed in tumor cells, it triggers a negative feedback loop that inhibits T cell activation, preventing them from recognizing and attacking tumor cells. This allows tumors to evade immune detection and enhance the activation of regulatory T cells, helping maintain immune homeostasis and prevent autoimmunity. Monoclonal antibodies targeting PD-1/PD-L1 block this interaction, restoring T-cell-mediated anti-tumor immunity and enabling tumor cell destruction 8,9. The 5-year survival rate of patients with advanced LUAD treated with PD-1/PD-L1 ICI monotherapy significantly improved compared with that of patients treated with previous chemotherapy 10. However, among patients with lung cancer receiving immunotherapy as first-line treatment, 7-27% develop primary drug resistance 11, and approximately 25% develop secondary drug resistance 12. The mechanisms of resistance to PD-1/PD-L1 ICI in patients with LUAD are complex and can be roughly divided into endogenous and exogenous factors. Endogenous resistance occurs due to cancer cell changes related to immune recognition, cell signaling, gene expression, and DNA damage response, while exogenous resistance occurs during the entire process of T-cell activation outside tumor cells 10. Factors such as low immunogenicity, unfavorable chemokine environment, regulatory T-cell activity, and endothelial growth factors significantly reduce the effectiveness of immunotherapy in treating LUAD. Therefore, identifying key molecular mechanisms of immune escape and the sources of resistance and heterogeneity in patients with LUAD to PD-1/PD-L1 drug therapy is crucial. Such research can help avoid overtreatment of early tumors and contribute to the development of individualized precision therapy. Identifying key regulatory molecules involved in immune escape also provides important targets for clinical prediction and treatment.

Single-cell sequencing analysis, including single-cell whole-genome sequencing and single-cell transcriptome sequencing (scRNA-seq), reveals changes in the genome and transcriptome, making it one of the most popular technologies in the field of biology. With its high resolution, it can accurately analyze the composition of sample cells, reveal the gene structure and gene expression status of a single cell and reflect heterogeneity between cells. The single-cell sequencing process includes four main aspects: single-cell isolation; cell lysis, genomic DNA acquisition, and whole-genome amplification; sequencing; and data analysis 13,14. Current methods primarily use single-cell recognition based on barcodes; that is, a specific DNA sequence is added to each cell, and the sequence carrying the same barcode is regarded as coming from the same cell during sequencing 14-16. The continuous development of single-cell sequencing technology will play an increasingly important role in analyzing cell heterogeneity, revealing the relationship between cell populations in the microenvironment, tracking the occurrence and development of diseases, and providing technical support for personalized prevention and treatment 13,17. RNA sequencing (RNA-seq) offers unique advantages for high-throughput, unbiased studies using minimal samples, allowing for more accurate gene expression measurement and higher sensitivity in the quantification of rare variants and transcripts. Its advantages are all-encompassing and multilevel.

While immunotherapy, particularly the use of PD-1/PD-L1 inhibitors, has significantly advanced the treatment of LUAD, noting that there remain unresolved challenges, particularly related to drug resistance and treatment heterogeneity, is important. Current research has focused heavily on the mechanisms of immune checkpoint inhibition. However, less attention has been paid to the cellular heterogeneity within the tumor microenvironment that contributes to immune escape and therapy resistance. Furthermore, although the development of single-cell sequencing technologies has revealed promising insights into tumor biology, understanding how these findings could be applied to LUAD is still limited, specifically in the context of resistance to PD-1/PD-L1 inhibitors.

This study aimed to create a comprehensive cellular map of LUAD using scRNA-seq to uncover the mechanisms behind PD-L1 monoclonal antibody resistance and immune escape. The study findings could support personalized medicine and improve clinical strategies.

## Materials and Methods

### Proportions of cell types

The eight LUAD samples used in this study were collected from tumor tissues of hospitalized patients at Zhongshan People's Hospital. The inclusion criteria were as follows: patients aged 18 to 85 years with a diagnosis of localized early-stage LUAD that was pathologically confirmed; patients who had not received immunotherapy, chemotherapy, or radiotherapy prior to enrollment; and patients with good organ function and no serious complications or chronic diseases. Exclusion criteria included patients with severe immune deficiency (such as HIV infection or those undergoing immunosuppressive therapy), patients with autoimmune diseases, organ transplant recipients, and pregnant or breastfeeding women. All patients were required to sign an informed consent form prior to enrollment, confirming their understanding of the study's purpose and potential risks. This study included only patients undergoing tumor tissue removal for the first time and required a full clinical evaluation to ensure that the selected patients were representative and aligned with the study needs.

### Single-cell suspension preparation and taxa identification

After the operation, the cut tissue was immersed in a 1× phosphate-buffered saline (PBS) tube and moved to a super-clean table, where the fresh tissue was cleaned with 1× PBS and then cut into a tissue homogenate of approximately 0.5 mm^3^ in a Petri dish using sterile surgical scissors. All operations were carried out at a low temperature of 4 ℃. A final concentration of 10% collagenase and 10% pancreatin was added to the centrifuge tube, and the culture medium supplement system was incubated on a constant temperature shaking table at 37℃ for 30 min. After digestion, the cell suspension was filtered through a 40 μm cell screen on a super-clean table, and the filtered solution was collected at 4 ℃ at 300 × *g*. The cardiac supernatant was removed after 5 min, retaining the precipitation at the bottom of the tube. Suspension cells (1× PBS + 0.04% BSA) were added.

The auto-annotated results of SingleR were used to determine the proportion of various cell types in a single sample (top), different groups (bottom), immune/stromal cells (left), and total cells (right). SingleR achieved cell type annotation by calculating the correlation between the expression profile of the self-tested sample and the reference sample. For this project, the software's built-in HumanPrimaryCellAtlasData reference dataset was used for the corresponding label. The main information included the cell type label, each cell's calculated expression spectrum, and the Spearman correlation between the expression of each reference sample's spectrum. Correlation analysis was conducted only for differences in gene expression levels, and the levels of self-measured samples and reference samples were normalized by logcounts. The score of the reference cell type label was defined by the correlation coefficient, and the label with the highest score was taken as the annotation result for the corresponding cell. Finally, the proportion of cells contained in each cluster corresponding to the annotated cell type was counted. The cell type label with the highest proportion was taken as the final cell type annotation result for the cluster.

The parameters are as follows: clusters = NULL, genes = 'de', de.method = 'classic', assay.type.test = 'logcounts', assay.type.ref = 'logcounts', no parameters specified, run with default parameters. These proportions are displayed in bar chart format using the ggplot2 package (version 3.3.5).

### Uniform Manifold Approximation and Projection (UMAP) plot

UMAP clustering results from Seurat (version 4.1.0) were labeled based on tumor malignancy predictions (malignant, normal, and undefined cells) derived from CopyKAT (version 1.1.0). The results were assigned a naming label in the legend. Clusters with more than 60% PD-L1-negative cells were labeled as PD-L1-negative; those with more than 60% PD-L1-positive cells were labeled as PD-L1-positive. Other clusters were marked as “unknown” in the UMAP plot and identified accordingly in the legend.

### Proportion of each cell cluster

The proportion of cells in each cluster was calculated using Seurat (version 4.1.0) based on tumor malignancy prediction results from CopyKAT and PD-L1 status (positive/negative). These proportions are shown in bar plot form using ggplot2 (version 3.3.5).

### Heatmap of Gene Set Variation Analysis (GSVA) enrichment score

To estimate pathway activity for each cell cluster, GSVA was performed using standard settings in the GSVA R package (version 1.32.0). A gene set comprising 50 hallmark pathways (h.all.v2023.1. Hs.symbols.gmt) was downloaded from the Gene Set Enrichment Analysis website (https://www.gsea-msigdb.org/gsea/index.jsp).

### Volcano plot

Differential expression analysis was conducted between PD-L1-positive and negative groups using the FindAllMarkers function in Seurat (version 4.1.0) with the parameters “min.pct = 0.25, thresh.use = 0.25”, plotted by ggplot2 (version 3.3.5). The top 10 markers were visualized using the DotPlot function. Additionally, significantly differentially expressed genes were identified based on avg_log2FC >1 and abs (pct.1-pct.2) >0.1.

### Pseudotime plot of normal cells

Pseudotime analysis was performed on cells with different PD-L1 statuses within normal cell clusters from Seurat (version 4.1.0) using Monocle 2 (version 2.22.0, http://cole-trapnelllab.github.io/monocle-release) with DDR-tree and default parameters.

### Radiation plot

Based on pseudotime analysis, branch expression analysis modeling (BEAM) was performed to determine branch fate using the BEAM function of Monocle 2 (version 2.22.0). Pseudotime BEAM genes were also calculated using p- and q-values to adjust for the significance of gene expression. Significant genes (q-value <0.0001) were selected for further analysis.

### Histogram and violin plots

Histograms and violin plots showing the expression of selected markers in different groups were generated using ggplot2 (version 3.3.5) and the VlnPlot function in Seurat (version 4.1.0).

### Bubble plot

Selected markers were visualized using the DotPlot function in Seurat (version 4.1.0) with default parameters.

### Protein-protein interaction network analysis of selected markers

Protein-protein interaction (PPI) network analysis was performed using the STRING website tools (https://string-db.org/) with default parameters.

### Real-time quantitative polymerase chain reaction (RT-qPCR)

The lung cancer cell line A-549 (CCL-185) and lung cell line HPAEC (PCS-100-022) used in this study were purchased from ATCC (https://www.atcc.org/) and cultured in DMEM medium containing 10% fetal bovine serum at 37 ℃ and 5% CO_2_. Cells were transferred every 2-3 days; those in the logarithmic growth stage were used for the experiment. Cells were lysed with Trizol solution, and RNA was extracted. Reverse transcription of mRNA was performed using the PrimeScript™ RT reagent Kit with gDNA Eraser (Perfect Real Time) (Code No. RR047Q; TAKARA, Beijing, China). RT-qPCR was performed using cDNA obtained from reverse transcription. The procedure followed the protocol of the TB Green® Premix Ex Taq™ II (Tli RNaseH Plus) (Code No. RR820Q; TAKARA Bio). Reverse transcription and fluorescence quantitative PCR procedures were performed according to the manufacturer's instructions. Primers and sequences used for the analysis are listed in Tables 1 and 2.

## Results

### Immune landscape of lung cancer tumors

**Figure 1:**
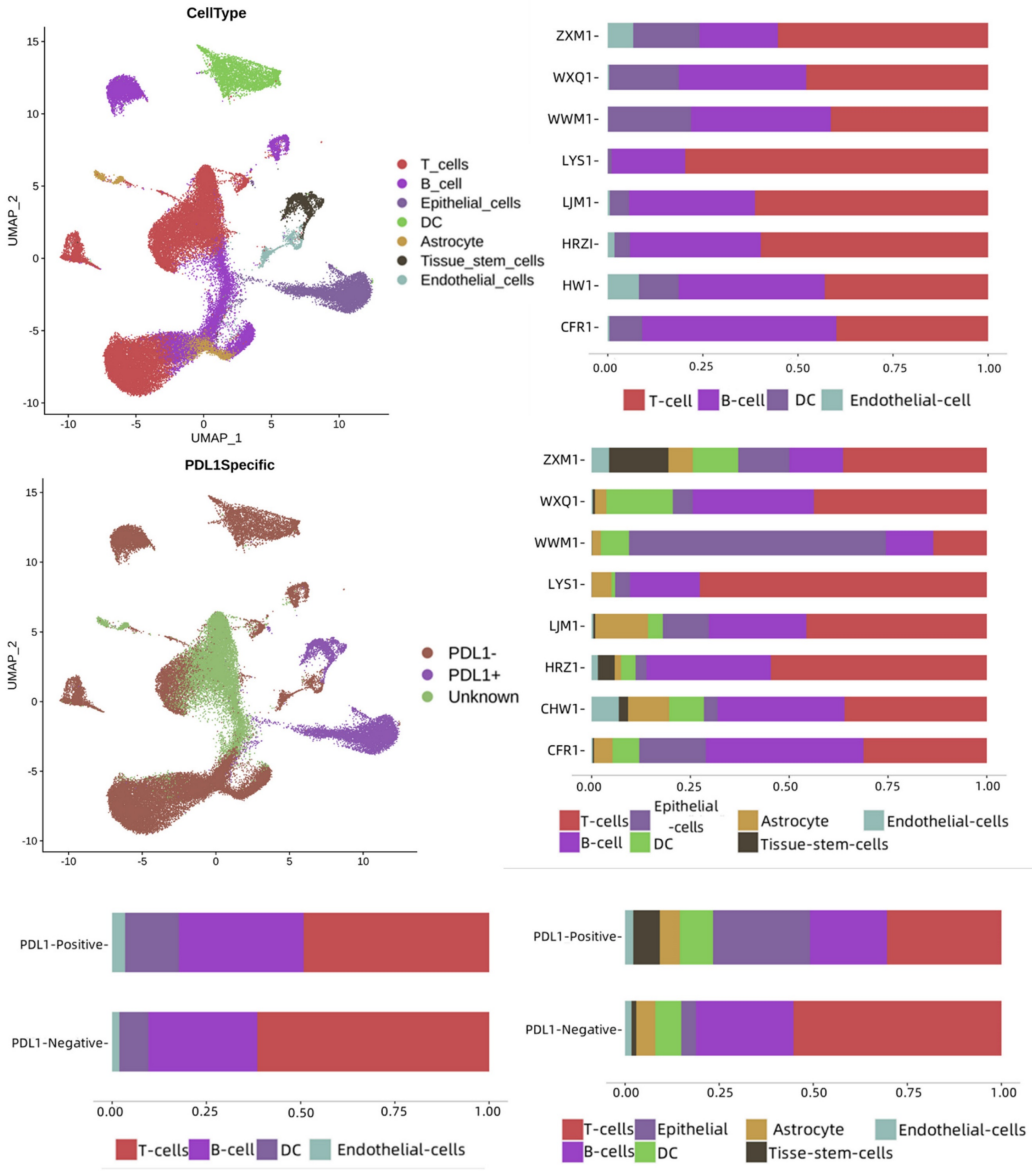
Single-cell landscape of lung cancer tumor and its PD-L1 immune signaling axis response. PD-L1: Programmed Death-Ligand 1

To assess the immune landscape based on the PD-L1 signaling axis and immune escape status, we collected tumor samples from eight patients with lung cancer for scRNA-seq and bioinformatics analysis. These samples included 4 without PD-L1 expression, 2 with PD-L1 expression, and 2 with high PD-L1 expression. In total, 58810 single cells were analyzed: 38,244 cells from four samples without PD-L1 expression, 14231 cells from two samples with PD-L1 expression, and 6335 cells from two samples with high PD-L1 expression. As shown in Figure 1, seven cell types were identified in the dataset: T cells, B cells, epithelial cells, DCs, astrocytes, tissue stem cells, and endothelial cells. These cell types were annotated separately for each group of cells and patients, and there were differences between PD-L1-positive and negative samples. T-cells and B-cells were dominant in both groups. The numbers of B cells, astrocytes, endothelial cells, epidermal cells, and tissue stem cells were higher in the PD-L1-positive sample group, whereas T cells and DCs were dominant in the PD-L1-negative sample group.

### Cell subpopulation analysis

**Figure 2:**
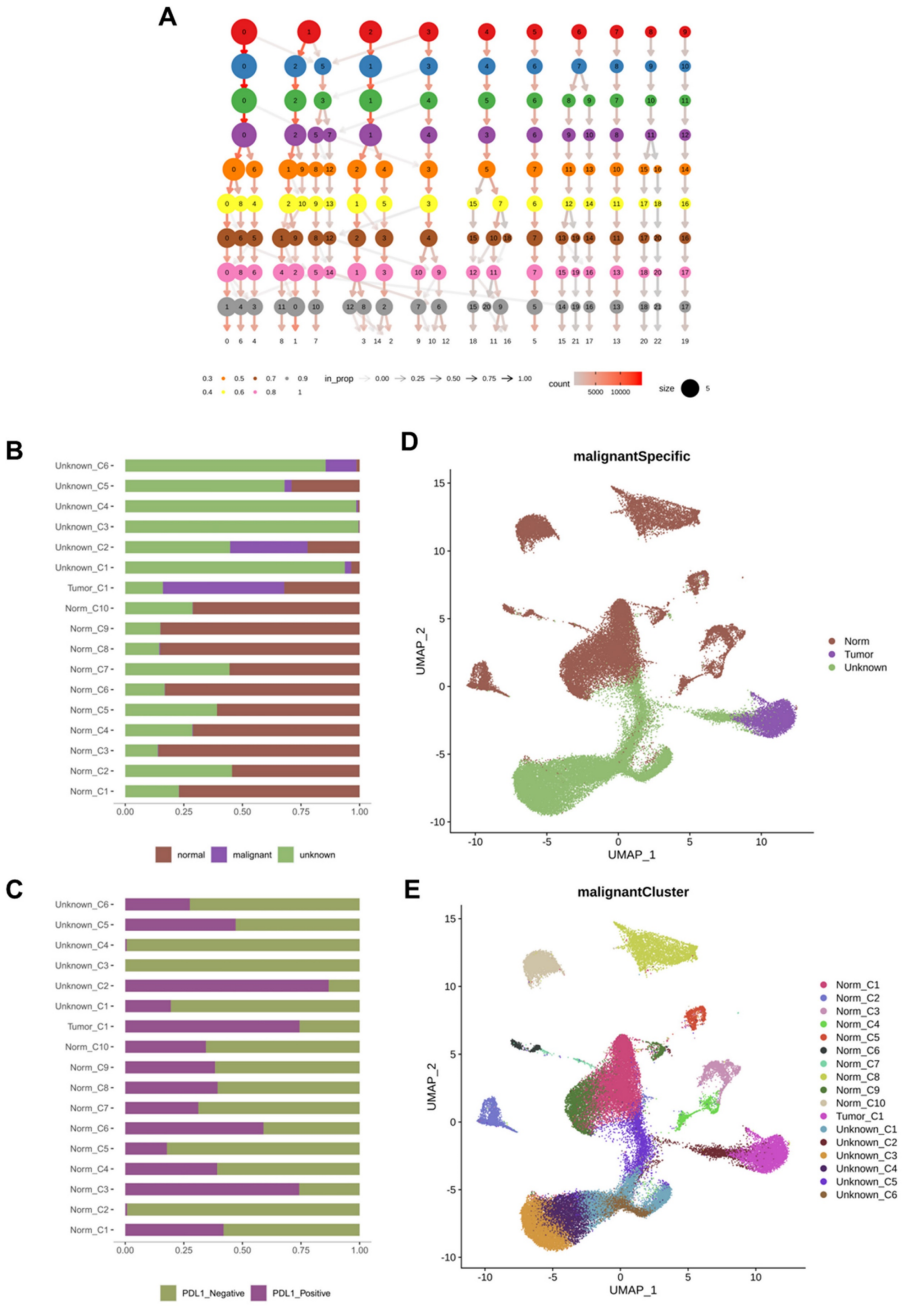
Cell subsets and their tumor marker responses. A: Cell subdivision map obtained by sequencing; B: The ratio of all cells to tumor cell markers; C: PD-L1 positive and negative ratio of cell subsets; D: UMAP of tumor, normal and unknown cell subsets; E: UMAP of different cell subpopulations. PD-L1: Programmed Death-Ligand 1; UMAP: uniform manifold approximation and projection

We further subdivided the seven cell types from different classes into 17 clusters (Figure 2A). Considering the large-scale chromosomal changes in cancer cells, we used molecular markers to annotate the 17 cell clusters as tumor or normal cells. Among the 17 clusters, one was identified as tumor cells, and another 6 clusters of cells were annotated as unknown; that is, it could not be determined whether they were tumor cells or normal cells (Figure 2B). To analyze the relationship between PD-L1 positivity and cell clusters, we compared the PD-L1 detection rates between different cell clusters (Figure 2C) and found that the positive rate of PD-L1 in the tumor_C1 cell cluster exceeded 50%. The negative rate of PD-L1 in most of the normal cell clusters was more than 50%, such as Norm_C1, Norm_C2, Norm_C4, Norm_C5, Norm_C7, Norm_C8, Norm_C9, and Norm_C10. The Norm_C3 and Norm_C6 clusters showed positive rates of PD-L1 of over 50%. According to the UMAP cluster analysis, tumor_C1 clusters were isolated from epithelial cells, and Norm_C3 and Norm_C6 clusters were isolated from stem cells and astrocytes, respectively (Figure 2D, E).

### PD-L1 pseudotime series analysis of normal cell communities

**Figure 3:**
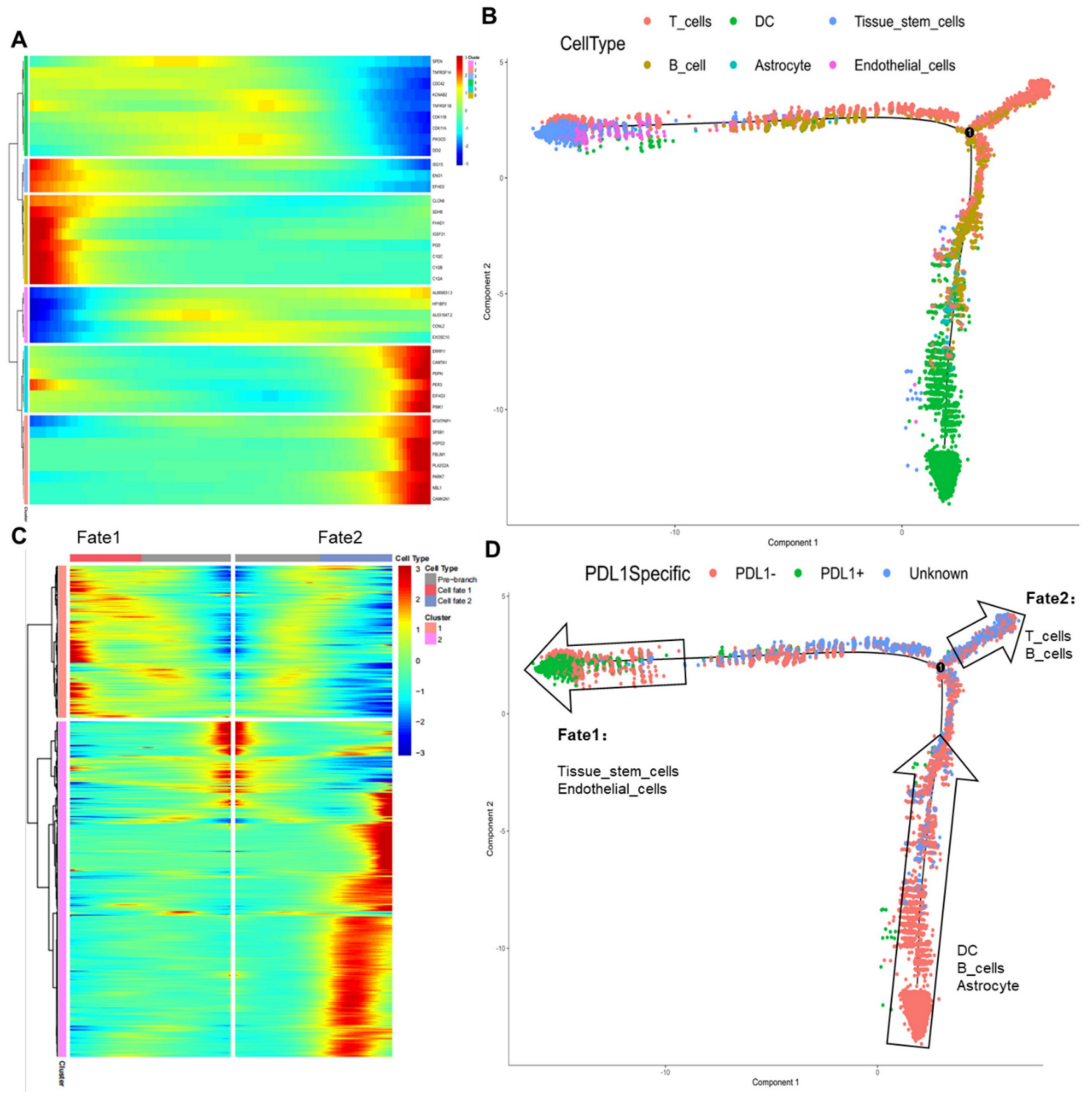
PD-L1 pseudotime of normal cell subsets. A: Classify and name the cells according to the time sequence; B: The position of 6 kinds of classified cells in the timeline; C: Cells were divided into Fate1 and Fate2 groups according to cell type. D: PD-L1 signal axis response and Fate1 and Fate2 cells were located in the time axis, respectively. PD-L1: Programmed Death-Ligand 1

The transcriptional differentiation trajectory of normal cells showed that the cells were divided into six different sequences according to the results of the different molecular markers (Figure 3A, B). By further comparing pseudotime results with PD-L1 labeling results (Figure 3C, D), we found that DCs, B cells, and astrocytes in the PD-L1-negative group differentiated into fate1 (PD-L1-positive group) or fate2 (unknown group). Initially, no PD-L1-positive DCs, astrocytes, or B cells entered the tumor tissue for an immune response. However, as the tumor progressed, a PD-L1-positive reaction gradually occurred after the differentiation of tissue stem cells and endothelial cells, resulting in immune escape. Meanwhile, immune cells, such as T and B cells with insignificant PD-L1-positive reactions, were retained.

### Molecular markers of differential genes in cells with different PD-L1 states

**Figure 4:**
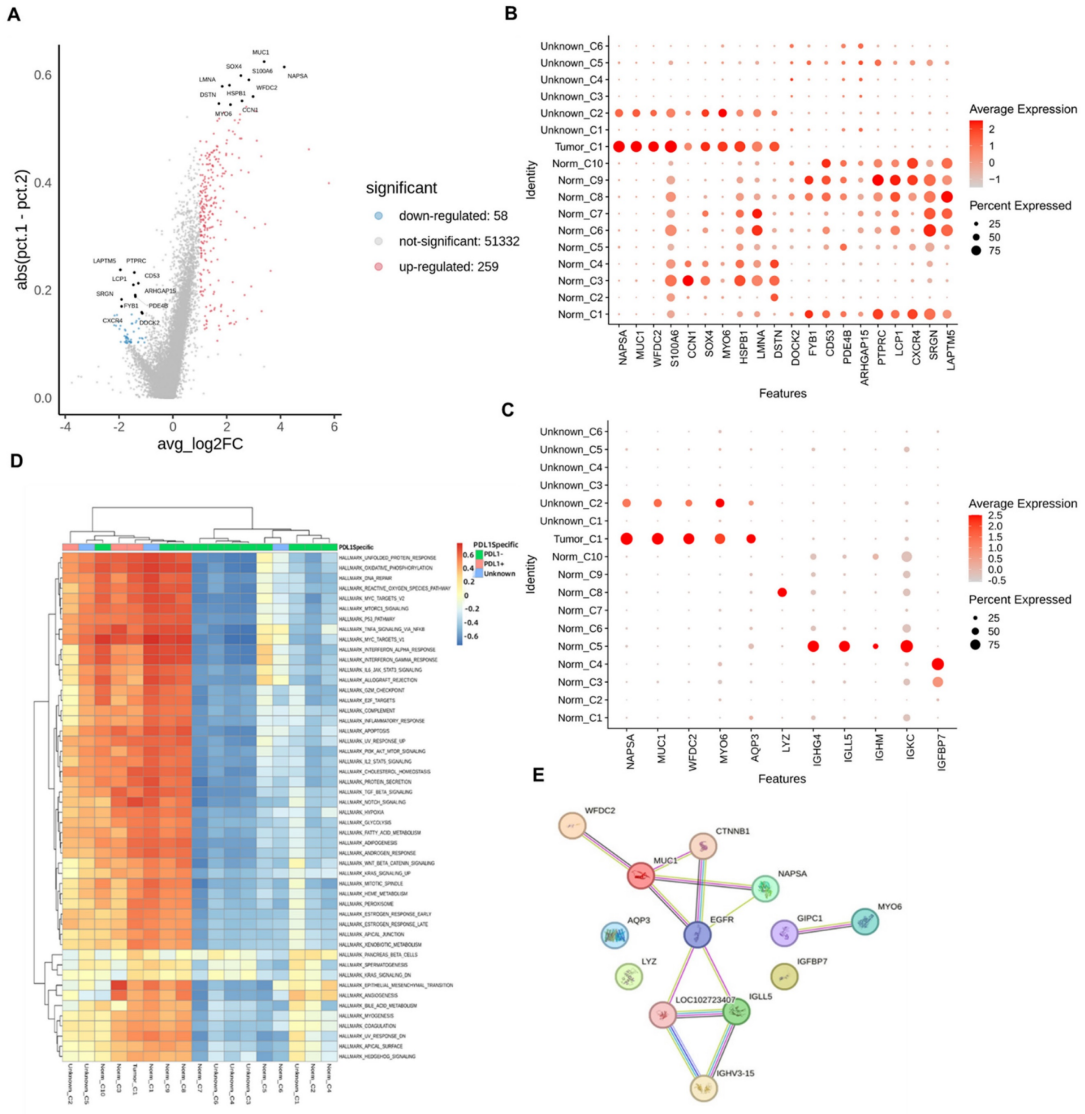
Molecular marker gene screening. A: Differential gene volcano map; B: Bubble map of expression of highly different genes in different cells; C: Bubble map of the expression of important genes in each cell; D: Differential channel heat map of different cell clusters; E: Screening important gene interaction networks.

In different cell clusters, genes with significantly different expression levels were found to be significantly higher than those in other cell clusters (Figure S1). The top 20 genes with the largest differences in different cells are shown in Figure 4A and B. According to Figure 4B, MUC1 in NAPSA, WFDC2, S100A6, CNN1, SOX4, MYO6, HSPB1, LMNA, DSTN, DOCK2, FYB1, CD53, PDE4B, ARHGAP15, PTPRC, LCP1, CXCR4, SRGN, and LAPTM5 were among the top 20. Among the genes with significant differences, NAPSA, MUC1, WFDC2, and MYO6 were well correlated with Tumor_C1 and Unknow_C2 (cells with a high positive rate of tumor markers), and these two cell clusters were all cells with a high positive rate of PD-L1. At the same time, MUC1 and AQP3 genes were also selected among Tumor_C1, Norm_C3, and Unknow_C2, which had a high positive rate of PD-L1, according to the bubble map (Figures S2-S7) to obtain more comprehensively correlated significant differential genes.

### MUC1, IGFBP7 gene and MYO6, IGFBP7 expression

We selected IGHG4, IGLL5, IGHM, and IGKC from nine normal cell clusters with high PD-L1 negativity rates. LYZ was selected in Norm_C8, and the characteristics of these genes were that their expression in the normal cell cluster was very different and significantly correlated with PD-L1-negative markers. In the PD-L1-negative cell cluster Norm_C2, the top ten differential genes were co-expressed in the PD-L1-positive cell cluster Norm_C3. Therefore, no genes significantly associated with negative PD-L1 expression were identified.

In conclusion, cells with high positive rates of PD-L1, NAPSA, MUC1, WFDC2, MYO6, AQP3, and IGFBP7 genes, with prominent differences in expression among Tumor_C1, Norm_C3, and Unknow_C2 were selected as PD-L1-positive molecular markers. Regarding cells with a high negative rate of PD-L1, we selected the IGHG4, IGLL5, IGHM, IGKC, and LYZ genes in Norm_C5, Norm_C8, and Norm_C2 as negative molecular markers to explore PD-L1 expression (Figure 4C). The expression levels of genes in different cells are presented in Table 3.

Pathway enrichment analysis of significantly differentially expressed genes was performed. As shown in Figure 4D, many pathways showed significant differences between PD-L1-positive and negative cells, with increased expression in PD-L1-positive cells, while decreased expression was seen in negative cells. Not all cells exhibited a consistent trend. Most of the differential signal transduction pathways are biologically based and involve relatively upstream genes. This indicates that the PD-L1 immune escape pathway is a complex and variable process in tumor tissues and that both tumor promotion and tumor suppression reactions occur and interact with each other.

A regulatory network analysis was performed on the selected 11 marker genes; the results are shown in Figure 4E. Among the 11 genes, PD-L1-positive molecular markers NAPSA, MUC1, and WFDC2 mainly act through EGFR receptors, and the PD-L1-negative molecular marker IGLL5 acts similarly. PD-L1-positive molecular markers MYO6, AQP3, IGFBP7, and PD-L1-negative molecular markers IGHG4, IGHM, IGKC, and LYZ acted through other pathways, ultimately affecting the expression or response of PD-L1. According to the box (Figure S8) and the violin (Figure S9) plots, in the PD-L1-positive and negative cells, AQP3, MUC1, MYO6, NAPSA, and IGKC produced significant differences, among which the expression levels of AQP3, MUC1, MYO6, and NAPSA genes in PD-L1-positive cells were significantly higher than those in negative cells. However, the expression levels of the IGKC gene in PD-L1-positive cells were significantly lower than those in negative cells, which was consistent with the results in different cell clusters.

### *In vitro* validation

**Figure 5:**
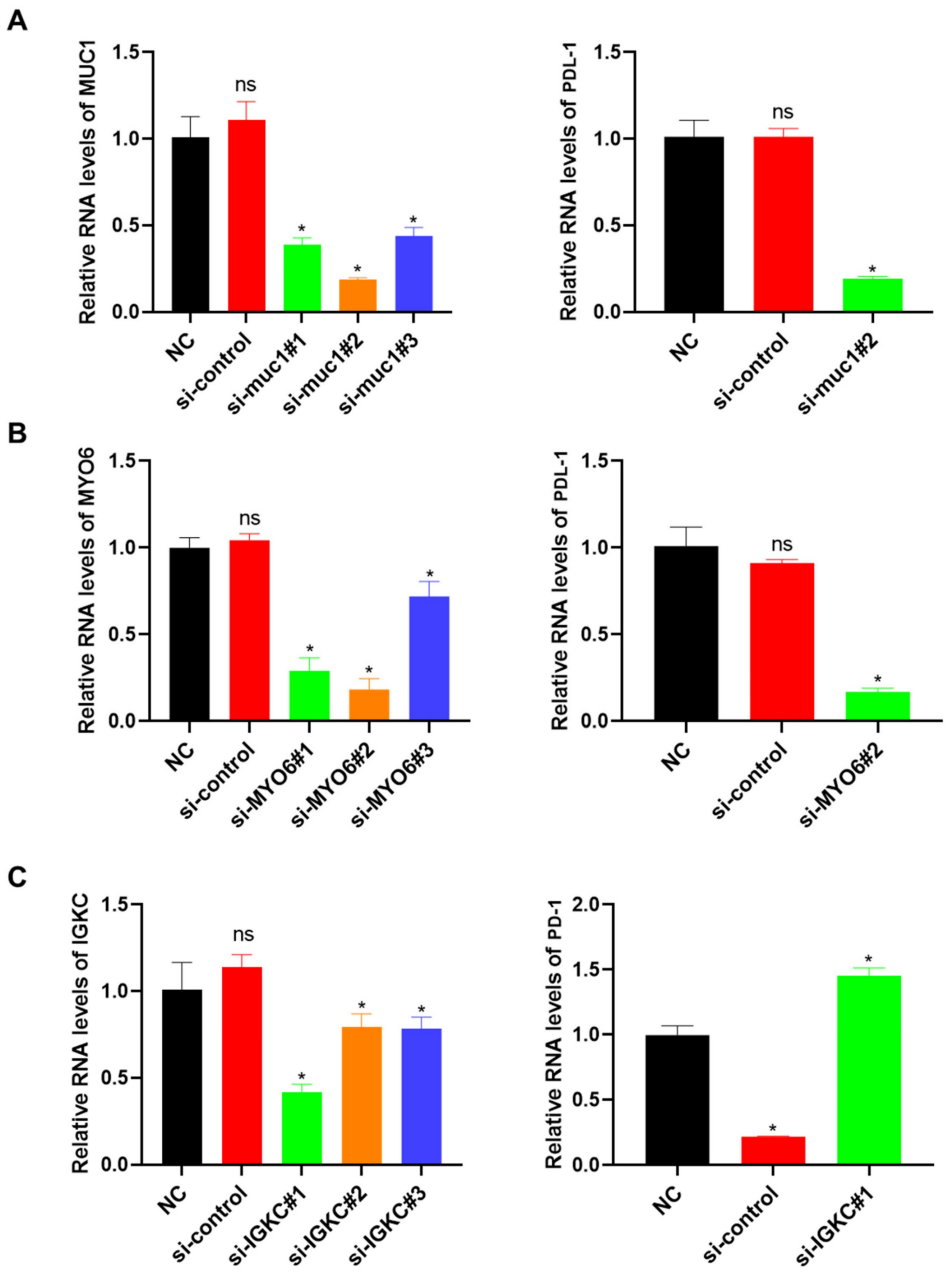
Verification of the relationship between candidate genes and the activation of PD-L1 immune signal axis response. A: Construction of MUC1 gene knock-down transient cell line and its effect on PD-L1 expression; B: MYO6 knockdown transient cell line construction and its effect on PD-L1 expression; C: Construction of IGKC gene knock-down transient cell line and its effect on PD-1 expression. PD-L1: Programmed Death-Ligand 1

To validate the sequencing results, we selected three genes (MUC1, MYO6, and IGKC) among the 11 important molecular marker candidates for silent transient cell line construction and transferred silent MUC1 and MYO6 into lung cancer cell line A-549, and silent IGKC into normal lung cell HPAEC. PD-L1 and PD-1 expression in each cell line was detected after the transient strain was obtained. As shown in Figure 5A, the si-muc1#2 cell line exhibited the lowest MUC1 expression among the three transient strains, leading to a significant reduction in PD-L1 expression. Similarly, in the si-MYO6#2 cell line, the lowest MYO6 expression resulted in decreased PD-L1 expression (Figure 5B). When the IGKC gene was silenced, the expression of PD-1 in SI-IGKC #1 normal lung tissue cell lines with the lowest expression level was significantly higher than that in the negative and positive controls, indicating that the PD-1 and PD-L1 immune escape signaling axes were activated with a decrease in IGKC expression level (Figure 5C).

## Discussion

In our study, we analyzed eight tumor samples from patients with lung cancer using scRNA-seq and bioinformatics. PD-L1 expression was absent in four cases, present in two, and highly expressed in the other two cases. The tumor's immune cells primarily consisted of T cells, B cells, epithelial cells, DCs, astrocytes, tissue stem cells, and a minor fraction of endothelial cells. We categorized these into tumor cells, normal cells, PD-L1-positive cells, and PD-L1-negative cells and examined the expression of differentially expressed genes. This analysis yielded eleven candidate marker genes, whose synergies with the PD-L1 immune escape signaling axis were confirmed.

From a cellular perspective, although the composition of PD-L1-positive and negative samples was analogous, the proportions of various cell types differed. In the PD-L1-positive sample group, there was a higher presence of B cells, astrocytes, endothelial cells, epidermal cells, and tissue stem cells, whereas the PD-L1-negative sample group exhibited a greater abundance of T cells and DCs. Through quasi-time series analysis, it was observed that DCs, astrocytes, and B cells lacking a PD-L1-positive response appeared to play a role in the early immune response to tumor tissue. However, immune escape occurred following tissue differentiation in later stages. Concurrently, immune cells such as T cells and B cells, showing no significant PD-L1-positive reaction, were preserved. This suggests that an immune escape reaction may transpire in the late stages of tumor growth, diminishing the efficacy of the immune cells' attack on tumor cells with increasing immune infiltration, thereby facilitating the formation and proliferation of lung tumors. Accordingly, the exploration of gene regulation furnishes insights into developing drugs to inhibit immune escape.

Immunotherapy has emerged as a potent clinical strategy for cancer treatment. Recent studies have shown a renewed interest in immunotherapy for managing patients with ICI-positive lung cancer. However, determining which patients with lung squamous cell carcinoma would benefit from immunotherapy remains challenging 18. The RNA-seq technique is an effective tool for exploring tumor heterogeneity and different cellular subpopulations, crucial for identifying potential therapeutic targets 19. We identified 11 marker genes associated with the PD-L1 immune escape signaling axis, including MUC1, IGKC, IGHG4, LYZ, IGLL5, and IGHM, all linked to immune responses. MUC1, a potent lipopeptic immune activator, is involved in specific immune responses and has been developed and utilized as a cancer vaccine 20-23. IGKCs play a significant role in the immune microenvironment of various tumors 24-26. Although less explored, IGHG4 is suggested to be involved in immune and transcriptional disorders, such as those observed in autistic dissonant identical twins, and may play a role in lymphocyte interactions 27,28. LYZ is markedly expressed in ovarian and estrogen-related immune responses and prominent in the immune invasion of diseases such as diabetic nephropathy and thyroid cancer 29-32. IGLL5 has been found to be involved in the immune invasion of renal cell carcinoma 33. IGHM activates the immune response in megakaryocytes, T cells, and other immune cells, subsequently triggering autophagy 34-36. This response is primarily related to the immune cell response during immune escape checkpoint sequencing. Additionally, genes like NAPSA, related to surfactant metabolism and autophagy, were identified 37,38. Significant differences were observed in the injury response, EGFR response factor WFDC2 39,40, transport-related factor MYO6 41, redox-regulatory factor, inflammatory damage response factor AQP3 42,43, and cell senility-related factor IGFBP7 44,45, all of which correlated with the PD-L1 response. These findings indicate that the PD-L1 response process is intricately linked to the immune system and involves complex processes related to cell metabolism, autophagy, injury, transport, reoxidation, aging, and others, a fact further confirmed by the enrichment analysis in our study.

Possible limitations of this study include a relatively small sample size consisting of only eight tumor samples, which may not have adequately represented the full heterogeneity of LUAD. Moreover, the cross-sectional design of the study might constrain the ability to monitor temporal changes in PD-L1 signaling and immune escape mechanisms. Additionally, potential biases in sample collection and processing could influence the generalizability of the findings. Further validation with larger, more diverse cohorts is required to corroborate these results.

In conclusion, this study indicated that a PD-L1 immune escape checkpoint response might be evident in the later stages of tumor tissue development. Eleven target genes—NAPSA, MUC1, WFDC2, MYO6, LYZ, IGHG4, IGLL5, IGHM, IGKC, AQP3, and IGFBP7—were identified for potential use in detecting and treating LUAD immune escape. Among these, three genes (MUC1, MYO6, and IGKC) have been experimentally validated for clinical application. Future research should focus on elucidating the functional mechanisms of PD-L1-mediated immune escape in LUAD, assessing the therapeutic efficacy of targeting these marker genes and evaluating the combined use of PD-L1 inhibitors with other treatments. Moreover, longitudinal studies are essential for monitoring the progression and resistance patterns associated with PD-L1 signaling.

## Supplementary Material

Supplementary figures.

## Figures and Tables

**Table 1:** Gene silencing sequence

Name	SS Sequence	AS Sequence
si-MUC1#1	GUUUUUGCAGAUUUAUAAACA	UUUAUAAAUCUGCAAAAACAU
si-MUC1#2	GCUUACAGUUGUUACGGGUUCUGGU	ACCAGAACCCGUAACAACUGUAAGC
si-MUC1#3	GGCCAGGAUCUGUGGUGGUACAAUU	AAUUGUACCACCACAGAUCCUGGCC
si-MYO6#1	AAUAUCGAGCUGAAGCCUGCAUUAA	UUAAUGCAGGCUUCAGCUCGAUAUU
si-MYO6#2	CGAGUAAGUUUGACCACAAGA	UUGUGGUCAAACUUACUCGAA
si-MYO6#3	GGAUCUGUGUUCAAGGCAAAG	UUGCCUUGAACACAGAUCCUA
si-IGKC#1	GGACCAAGCUGGAGAUCAAAC	UUGAUCUCCAGCUUGGUCCCC
si-IGKC#2	GCAAAGCAGACUACGAGAAAC	UUCUCGUAGUCUGCUUUGCUC
si-IGKC#3	GAGCAGGACAGCAAGGACAGC	UGUCCUUGCUGUCCUGCUCUG

**Table 2:** quantitative polymerase chain reaction primer sequence

Gene Name		Sequence	Long
MUC1	f	CGACGTGGAGACACAGTTCA	208bp
	r	CCAGACTGGGCAGAGAAAGG	
MYO6	f	CCCACTCCTAGAAGCCTTTGG	212bp
	r	CAGAAGCACCAGCACACAAC	
IGKC	f	AGTGGGTCTGGGACAGACTT	296bp
	r	CTCTCCTGGGAGTTACCCGA	

**Table 3:** Expression of marker genes in different types of cells

Gene name	highly expressed cells
NAPSA	Unknow_C2; Tumor_C1
MUC1	Unknow_C2; Tumor_C1
WFDC2	Unknow_C2; Tumor_C1
MYO6	Unknow_C2; Tumor_C1
AQP3	Unknow_C2; Tumor_C1
LYZ	Norm_C8
IGHG4	Norm_C5
IGLL5	Norm_C5
IGHM	Norm_C5
IGKC	Norm_C5
IGFBP7	Norm_C4; Norm_C3

## Abbreviations

BEAM: branch expression analysis modeling
CTLA-4: cytotoxic T lymphocyte-associated antigen-4
DCs: dendritic cells
GSVA: Gene Set Variation Analysis
PD-1: programmed cell death-1
PD-L1: programmed cell death-ligand 1
scRNA-seq: single-cell RNA sequencing
ICI: immune checkpoint inhibitor
LUAD: lung adenocarcinoma
MUC1: mucin 1
IGKC: immunoglobulin kappa constant
IGHG4: immunoglobulin heavy constant gamma 4
LYZ: lysozyme
IGLL5: immunoglobulin lambda-like polypeptide 5
IGHM: immunoglobulin heavy constant mu
NAPSA: napsin A aspartic peptidase
RNA-seq: RNA sequencing
RT-qPCR: real-time quantitative polymerase chain reaction
WFDC2: WAP four-disulfide core domain protein 2
MYO6: myosin VI
AQP3: aquaporin 3
IGFBP7: insulin-like growth factor binding protein 7
EGFR: epidermal growth factor receptor
UMAP: Uniform Manifold Approximation and Projection
