# Supplementary material for: Single-Cell Sequencing Reveals PD-L1-Mediated Immune Escape Signaling in Lung Adenocarcinoma
Source: J Cancer. 2025 Jan 27;16(5):1438–50. doi: 10.7150/jca.103656 (PMC11843243; doi:10.7150/jca.103656)
Supplement: Supplementary file 1 — Supplementary figures. [file jcav16p1438s1.pdf]

Supplementary

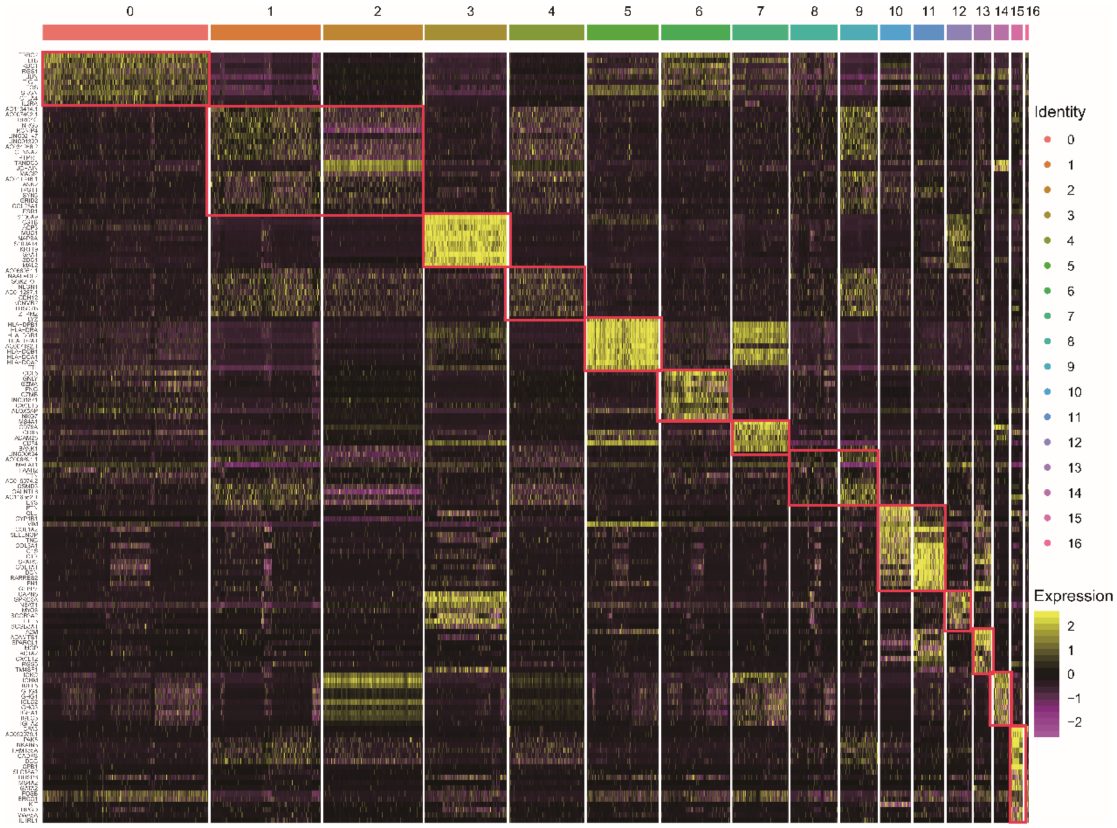

Figure S1 Top10.markersGene.DOHeatmap

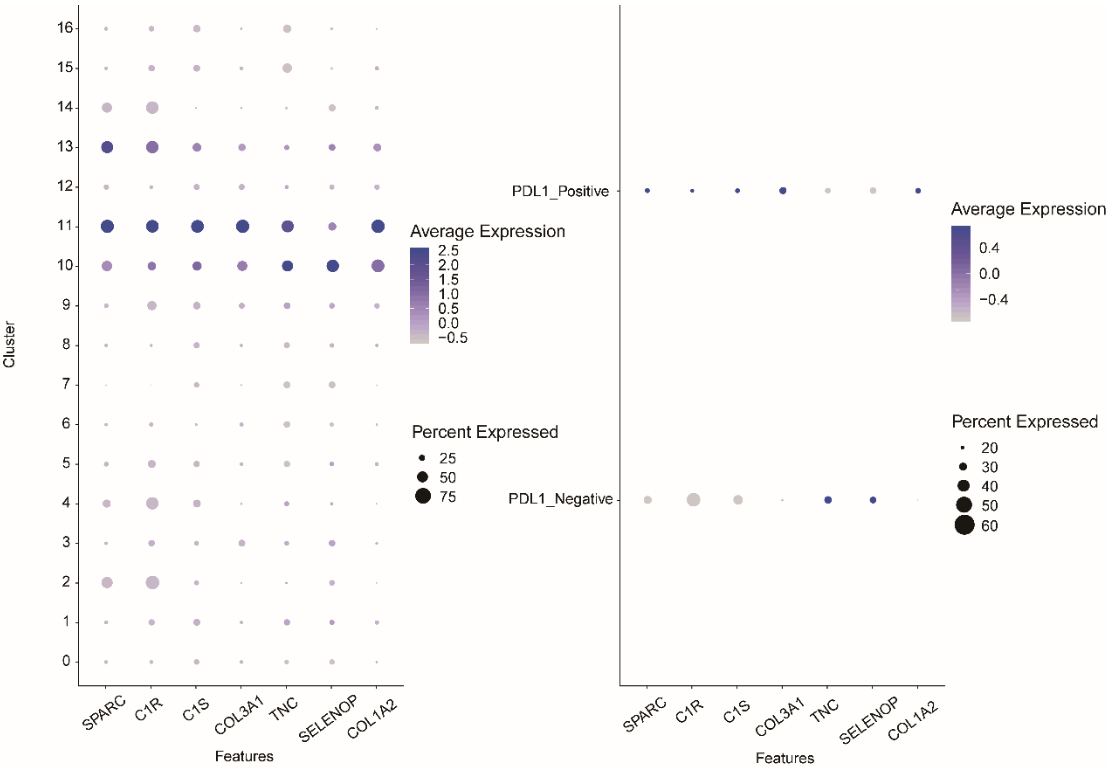

Figure S2 Norm_C2.Top10.markersGene.DotPlot

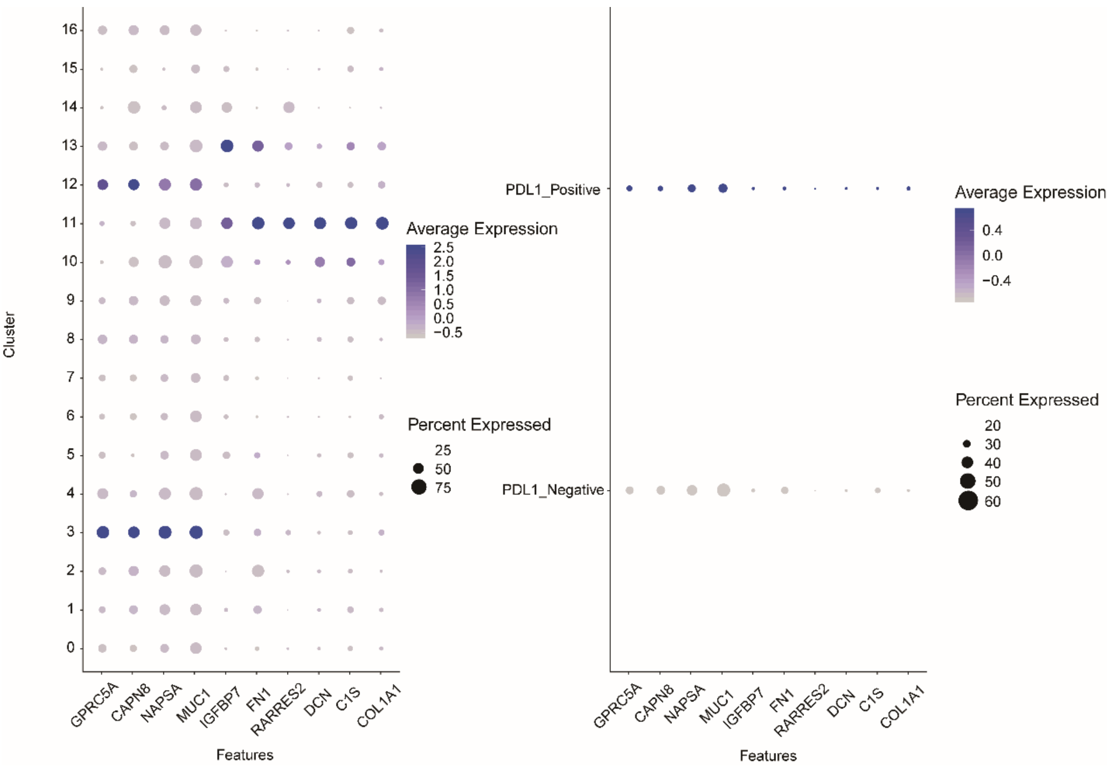

Figure S3 Norm_C3.Top10.markersGene.DotPlot

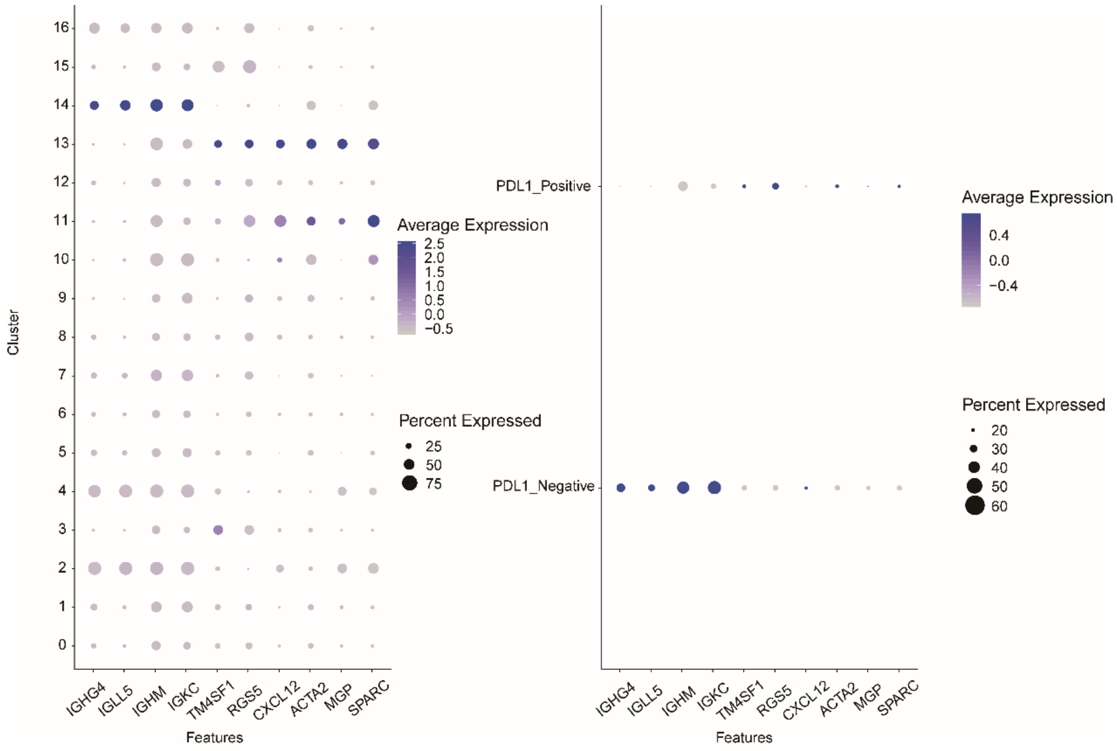

Figure S4 Norm_C5.Top10.markersGene.DotPlot

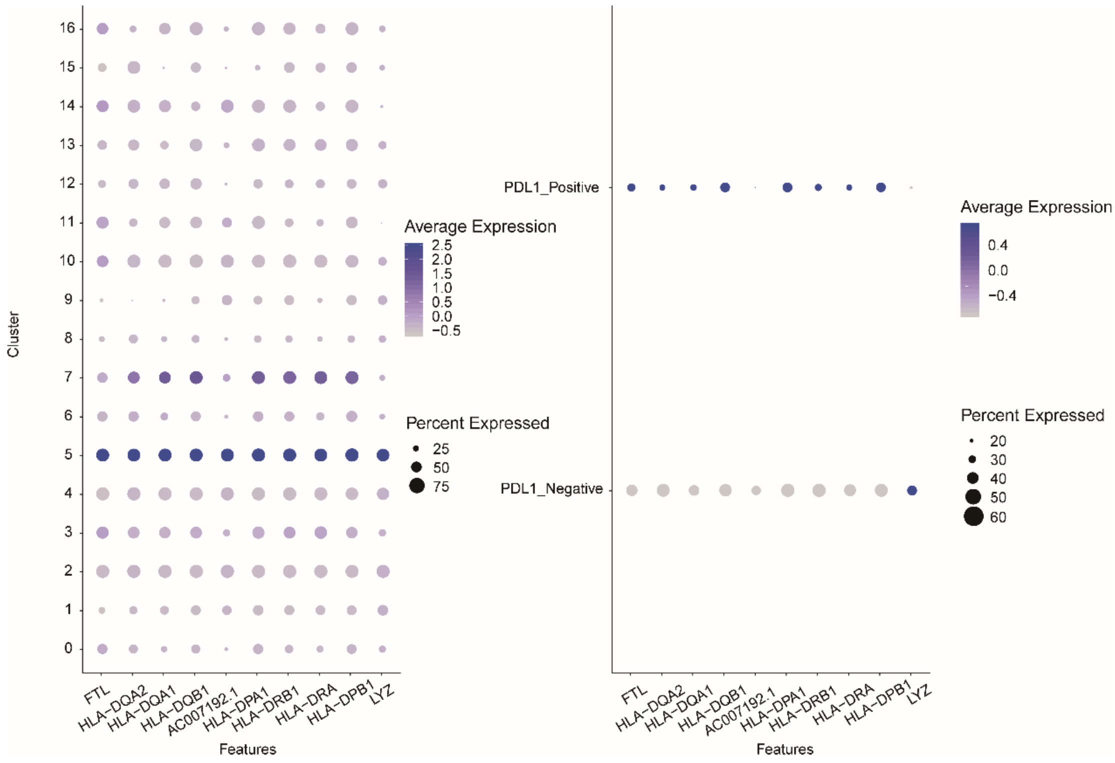

Figure S5 Norm_C8.Top10.markersGene.DotPlot

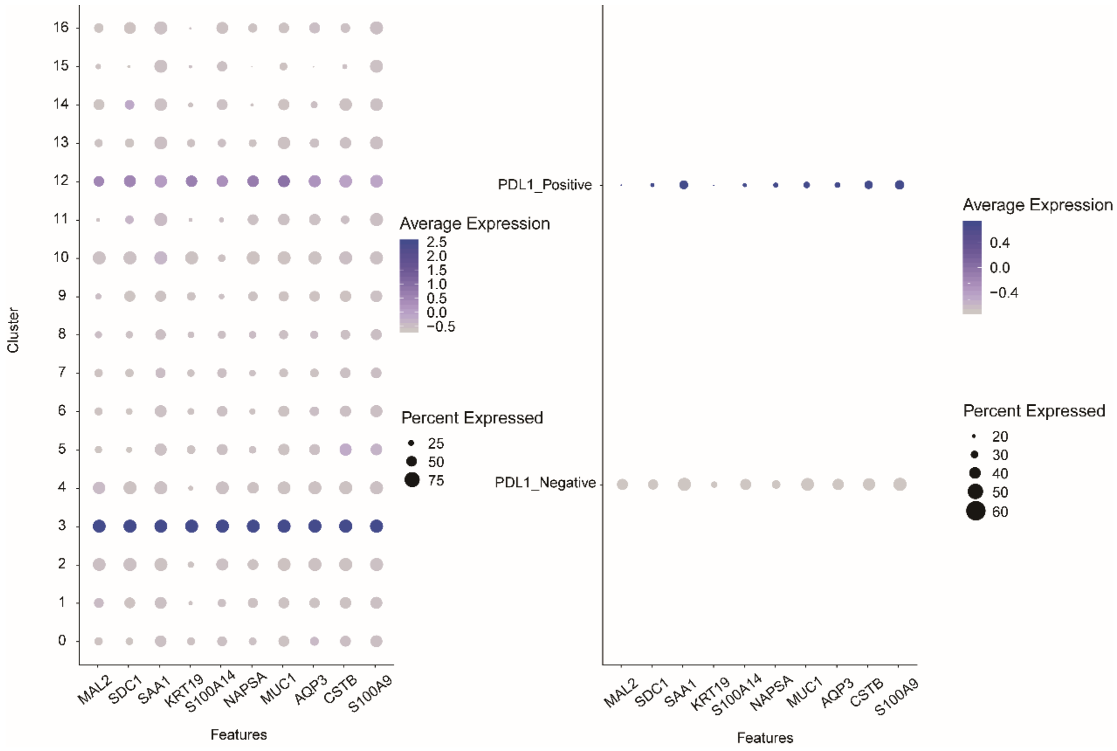

Figure S6 Tumor_C1.Top10.markersGene.DotPlot

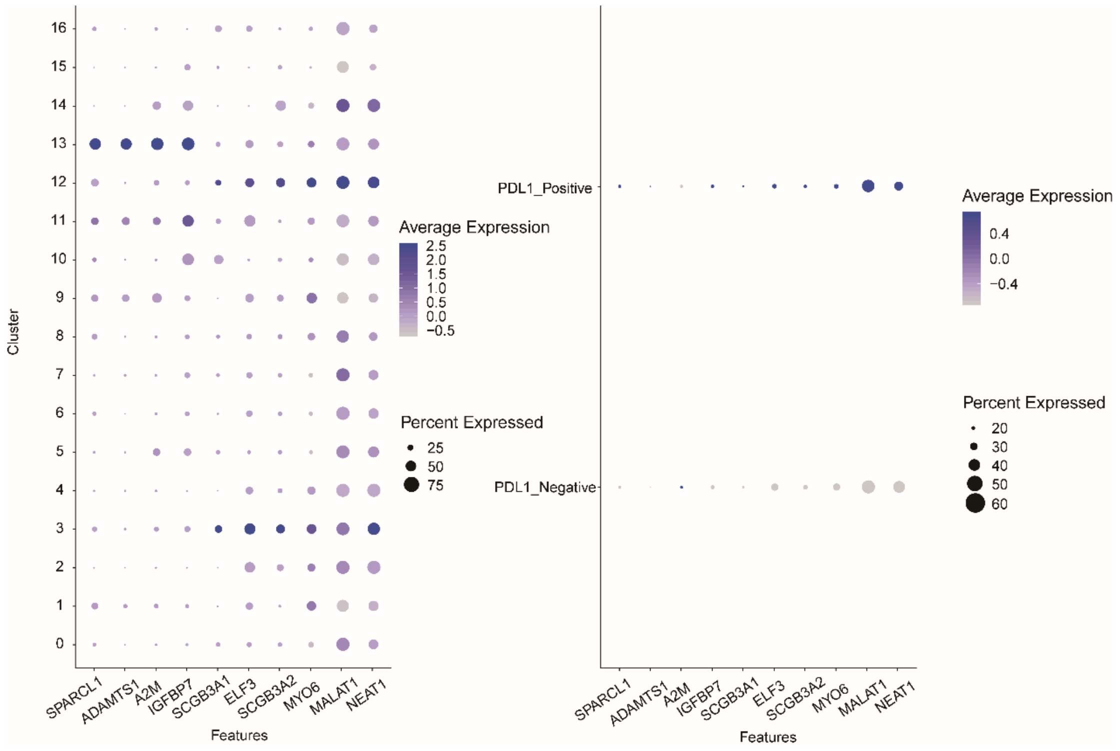

Figure S7 Unknow_C2.Top10.markersGene.DotPlot

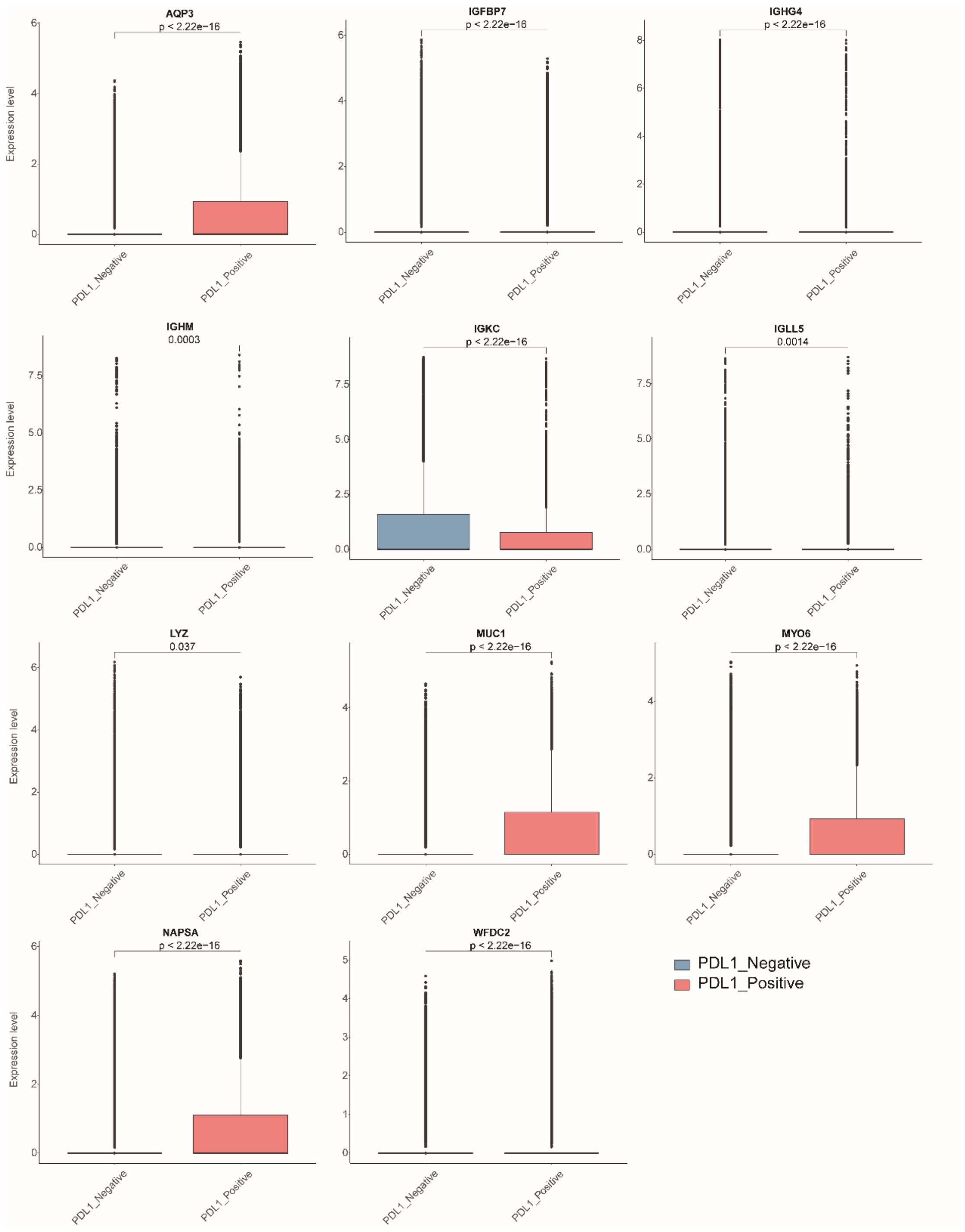

Figure S8 Boxplot

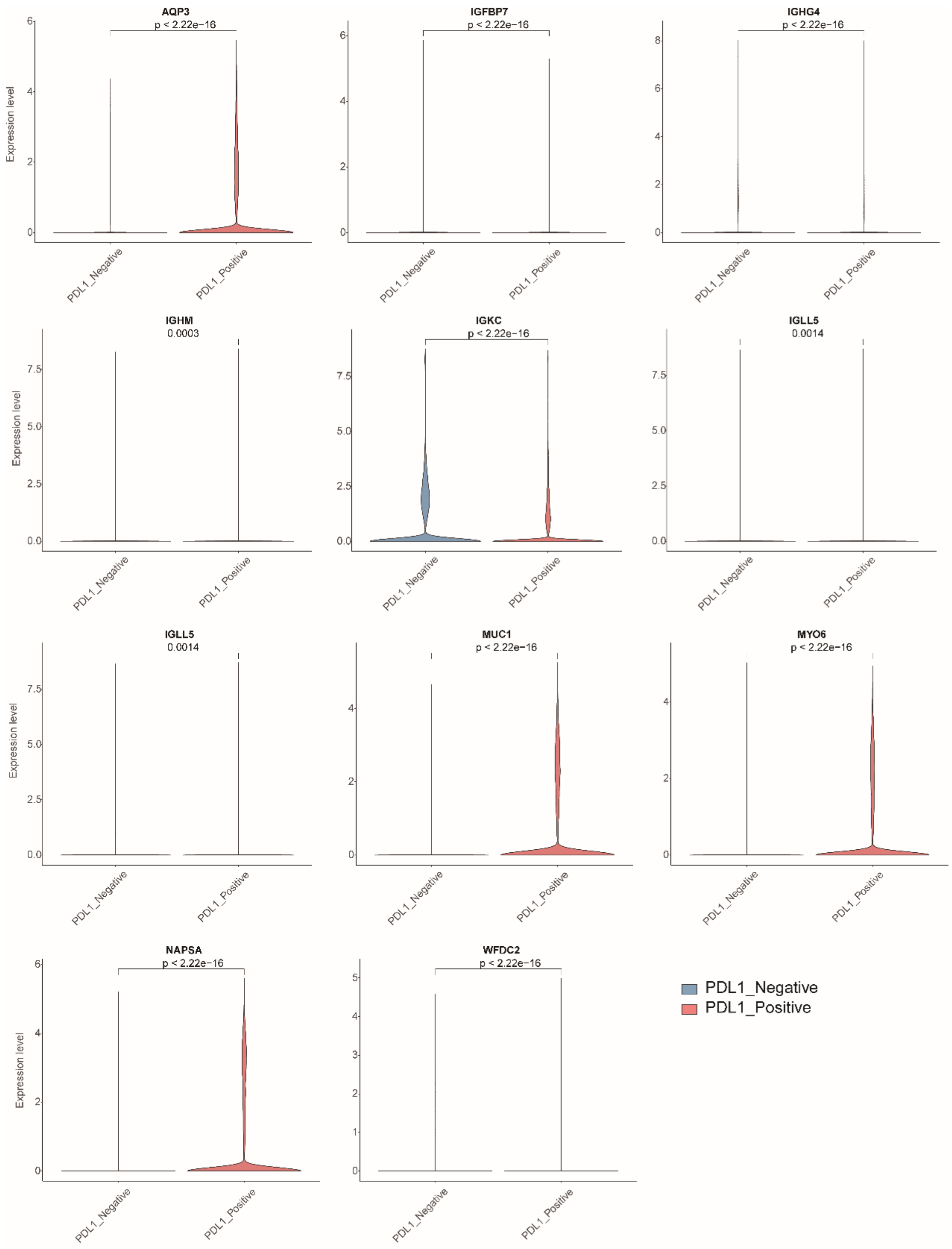

Figure S9 Vlnplot
